# Cofilin Activation during Podosome Belt Formation in Osteoclasts

**DOI:** 10.1371/journal.pone.0045909

**Published:** 2012-09-25

**Authors:** Anne Blangy, Heiani Touaitahuata, Gaelle Cres, Geraldine Pawlak

**Affiliations:** 1 Centre de Recherche de Biochimie Macromoleculaire, Montpellier University, CNRS UMR 5237, Montpellier, France; 2 Université Joseph Fourier, Grenoble, France; University of Toronto, Canada

## Abstract

Podosomes are dynamic actin-based structures found constitutively in cells of monocytic origin such as macrophages, dendritic cells and osteoclasts. They have been involved in osteoclast cell adhesion, motility and matrix degradation, and all these functions rely on the ability of podosomes to form supra-molecular structures called podosome belts or sealing zones on mineralized substrates. Podosomes contain two distinct domains, an actin-rich core enriched in actin polymerization regulators, surrounded by a ring of signaling and plaque molecules. The organization of podosome arrays into belts is linked to actin dynamics. Cofilin is an actin-severing protein that is known to regulate cytoskeleton architecture and cell migration. Cofilin is present in lamellipodia and invadopodia where it regulates actin polymerization. In this report, we show that cofilin is a novel component of the podosome belt, the mature osteoclast adhesion structure. Time-course analysis demonstrated that cofilin is activated during primary osteoclast differentiation, at the time of podosome belt assembly. Immunofluorescence studies reveal a localization of active cofilin in the podosome core structure, whereas phosphorylated, inactive cofilin is concentrated in the podosome cloud. Pharmacological studies unraveled the role of a specific cofilin phosphatase to achieve cofilin activation during osteoclast differentiation. We ruled out the implication of PP1/PP2A and PTEN in this process, and rather provided evidence for the involvement of SSH1. In summary, our data involve cofilin as a regulator of podosome organization that is activated during osteoclast differentiation by a RANKL-mediated signaling pathway targeting the SSH1 phosphatase.

## Introduction

Osteoclasts (OCs) are multinucleated cells of hematopoietic origin that degrade bone matrix. To perform this function, OCs must adhere firmly to bone using specialized adhesion structures called podosomes [1]. Podosomes are highly dynamic structures, containing an actin-rich core extending perpendicularly to the substrate, surrounded by a ring of associated proteins [2], [3]. The core of podosomes is enriched in several actin-associated proteins (Arp2/3, cortactin, WASp, WIP, dynamin, gelsolin) [2], which regulate actin polymerization [4]. Proteins present in the podosomal ring include integrins and various signaling and adaptor proteins among which vinculin and paxillin, the tyrosine kinases Src and Pyk2, and small GTPases of the Rho family. The actin cores undergo continuous polymerization and severing processes, and this dynamic was recently shown to play a critical role in regulating podosome stability in OCs [5].

Osteoclastic podosomes are highly dynamic and reorganize during OC maturation and activity [4]. Individual podosomes are connected to their neighbors by F-actin cables [6]. This allows podosome compaction to generate the different OC specific superstructures: podosome clusters and rings in immature OCs, the podosome belt at the periphery of mature OCs and the sealing zone when they resorb bone mineralized matrix [4], [7], [8]. Although podosomes are found in a large variety of myeloid cell types and also some epithelial cells [9], the supra-molecular organization of podosomes in belts and sealing zones is specific to OCs. In addition, these structures are essential for OCs to perform their function, as they have been involved in cell adhesion, migration, and bone resorption [9]. Pathways that regulate podosome patterning are therefore of importance in understanding bone resorption in both physiological and pathological conditions.

Cofilin is a key regulator of actin dynamics by stimulating the depolymerization and severing of actin filaments [10]. Multiple mechanisms have been identified that regulate cofilin activity [11], [12], among which phosphorylation [13]. Cofilin is phosphorylated at serine-3 by LIM- and TES-family kinases, which inhibits its ability to bind G- and F-actin, and to sever F-actin, thereby inactivating it [11]. Cofilin dephosphorylation and activation are mediated mainly by the Slingshot (SSH) family of phosphatases and the HAD-type phosphatase CIN [14]–[16]. Other phosphatases have also been involved, among which the Ser/Thr phosphatases PP1, PP2A and PP2B, and more recently PTEN [17]. Cofilin has been localized to invadopodia [18], [19], a structure related to podosomes, found in transformed cells [20]. In these structures, cofilin severing activity creates free barbed ends supporting Arp2/3-dependent actin polymerization [21]. Knockdown of cofilin by short interfering RNA (siRNA) led to a decreased life span of invadopodia in carcinoma cells [18]. The presence of cofilin in macrophage podosomes has been mentioned in a review [2], but no study has been published yet.

The aim of the present work was to investigate cofilin localization and activity in primary OCs. We established that cofilin is activated during OC differentiation then we analyzed the spatial and temporal regulation of this activation. Our main conclusions are that cofilin is addressed to podosome cores where it becomes activated at the time of belt assembly. This activation is mediated by a phosphatase under the control of RANKL, most probably SSH1.

## Materials and Methods

### Ethics Statement

Harvesting of murine bone marrow from sacrificed mice was approved by the regional ethic committee of Languedoc-Roussillon (France). Approval ID number: CEEA-LR-1054.

**Figure 1:**
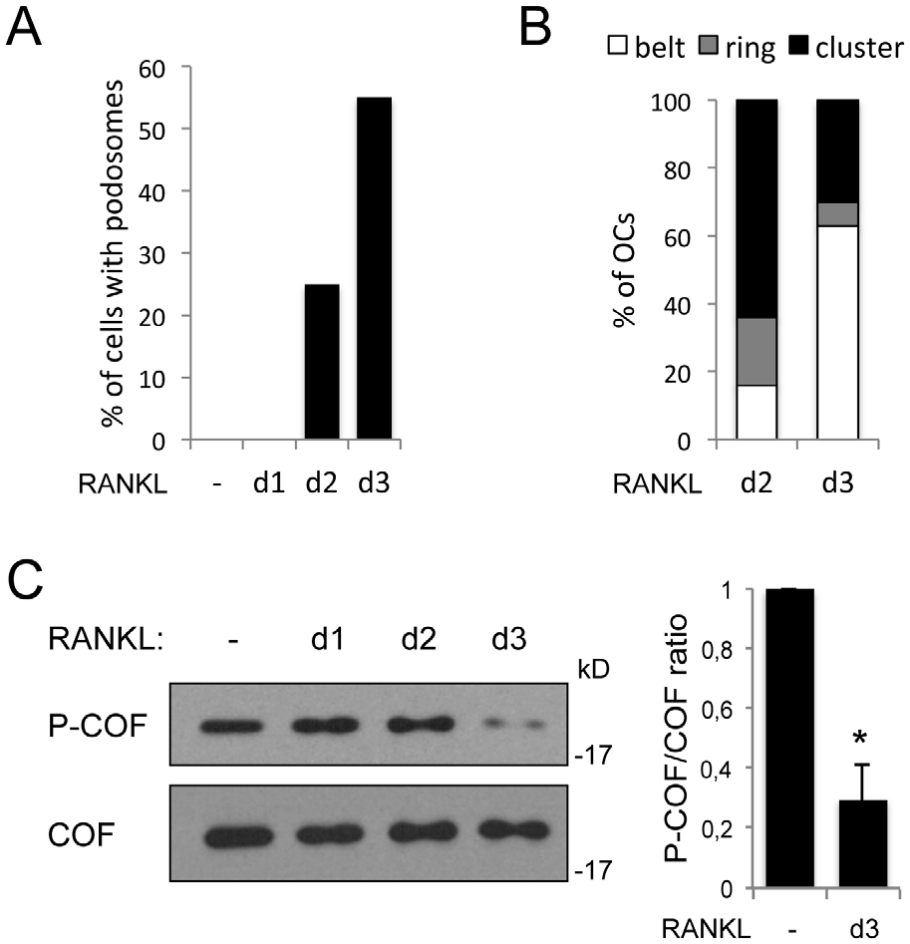
Cofilin is activated when podosome belts are assembled. BMMs were cultured for 3 days in the absence (−) or in the presence (d1 to d3) of RANKL. Each day, cells grown on coverslips were fixed and stained for actin, and cell lysates were prepared in laemmli sample buffer. Shown is a representative of 3 independent kinetics. (A) For each time point of the kinetic, the percentage of cells displaying podosomes was determined by counting at least 1000 cells per condition in random fields, under an upright microscope. (B) Podosome distribution in belts versus clusters and rings was analyzed in OCs (defined as cells with at least 3 nuclei) at day 2 and 3 of RANKL addition. The percentage of each category is represented. (C) Protein extracts were prepared and equal amounts of proteins were immunoblotted for phospho-cofilin (P-COF). Total cofilin was revealed after stripping of the phospho-cofilin antibody to serve as loading control. Right panel: densitometric analysis of the ratio of phospho-cofilin to total cofilin levels in unstimulated BMMs (−), versus OCs placed in RANKL for 3 days (d3) (*, *p*<0,0001, *t*-test, *n* = 6). Data represent the mean ± S.D.

### Isolation, Culture and Osteoclastic Differentiation of Mouse Bone Marrow-derived Macrophages

Bone marrow cells were purified from long bones of 4- to 8-week-old C57BL/6 mice sacrificed by cervical dislocation, as described [22]. Non-adherent bone marrow cells were cultured in αMEM containing 10% heat-inactivated fetal calf serum (Hyclone) and 2 mM glutamine, supplemented with 30 ng/ml M-CSF (Peprotech) for 48 h and used as bone marrow-derived macrophages (BMMs). For osteoclast differentiation, BMMs were cultured in the presence of 100 ng/ml RANKL and 30 ng/ml M-CSF (Peprotech), at the density of 6×10^4^ cells/well (12-well plate) or 1.5×10^5^ cells/well (6-well plate). Media was changed and cytokines were replenished every 2 days. OCLs generally appeared after 3 to 5 days. The term “pre-osteoclast” (pOC) refers to BMMs induced by M-CSF and RANKL for 2 days but still mononucleated. Alternatively, BMMs were maintained in M-CSF only as a control for undifferentiated cells.

**Figure 2:**
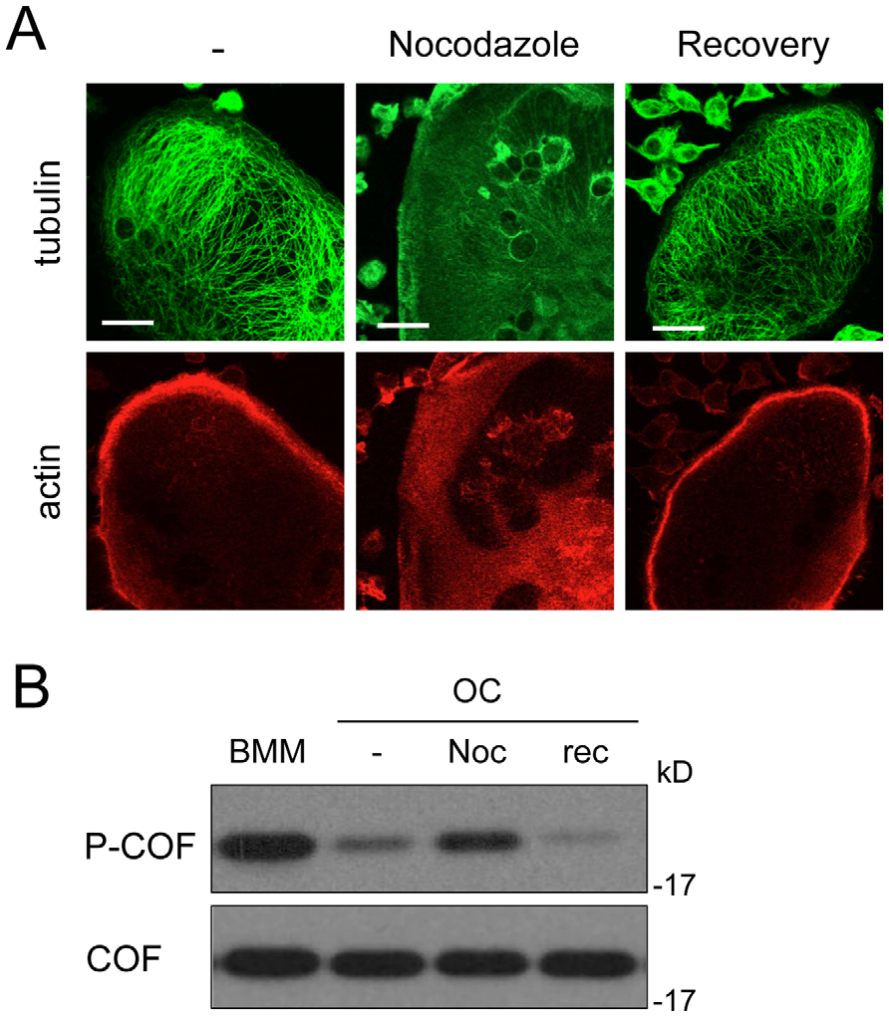
Podosome belt destabilization correlates with cofilin inactivation. OCs on day 4 of differentiation were treated for 30 min by 2 µM Nocodazole (Noc) or not (−) to depolymerize microtubules. Nocodazole was then washed-out and the cells were allowed to recover (rec) for 20 h at 37°C. (A) Cells plated on coverslips were viewed by confocal microscopy after fixation and labeling for microtubules (green) and F-actin (red). Note the collapse of both microtubules and podosome belts under nocodazole treatment and their reformation 20 h after washout (recovery). Scale bars: 10 µm. (B) Protein extracts were prepared and equal amounts of proteins were immunoblotted for phospho-cofilin (P-COF). Total cofilin was revealed after stripping of the phospho-cofilin antibody to serve as loading control. BMM extracts were loaded to show the initial level of phospho-cofilin.

### Antibodies and Reagents

Monoclonal antibodies to actin, tubulin, p190RhoGAP and vinculin, bisbenzimide Hoechst dye and TRITC labeled-Phalloidin were from Sigma. Other antibodies were as follow: monoclonal anti-cortactin (Millipore), polyclonal anti-cofilin (Cytoskeleton), polyclonal anti-phospho-cofilin (Cell Signaling Technologies), polyclonal anti-SSH1 (ECM Biosciences), polyclonal anti-GFP (Torrey Pines Biolabs). Alexa® Fluor 488- or 546-conjugated secondary antibodies were from Invitrogen. The microtubule disrupting agent nocodazole, the phosphatase inhibitor Na_3_VO_4_ and the ROCK inhibitor Y27632 (Y27) were purchased from Sigma, the PP1/PP2A inhibitor okadaic acid and the ROCK inhibitor H-1152 were from Calbiochem, and the PTEN inhibitor VO-OHpic was from Biovision.

**Figure 3:**
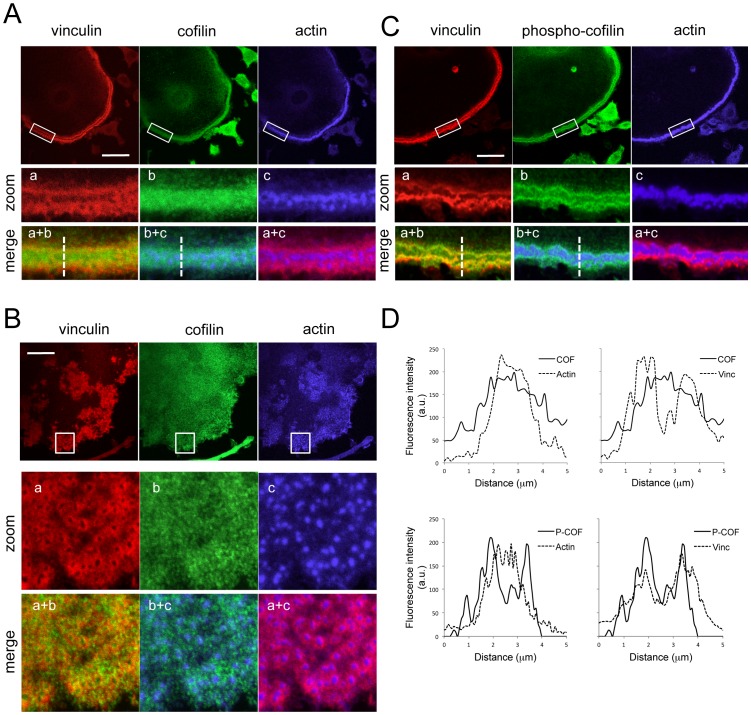
Spatial distribution of cofilin phosphorylation in podosome belts. (A) Distribution of vinculin (red), actin (blue), and cofilin (green) in OCs differentiated on glass coverslips and displaying mature podosome belts. Cells were observed and images were acquired by confocal microscopy. Images in lower panels are magnifications and merged images of the boxed regions in the top images. Scale bars: 10 µm. Note the colocalization of cofilin with both vinculin and actin. (B) Cofilin distribution was analyzed in OCs displaying podosome clusters as described in A. Scale bars: 10 µm. Note the absence of colocalization between cofilin and either vinculin or actin. (C) Phospho-cofilin distribution was analyzed in OCs displaying mature podosome belts as described in A. Scale bars: 10 µm. Note the colocalization of phospho-cofilin with vinculin, but not with actin. (D) Fluorescence intensity profiles of cofilin (COF) or phospho-cofilin (P-COF), together with actin or vinculin, measured across podosome belts (dashed lines in merged images from panels A and C). Each profile is a representative of at least 6 belts from three different differentiations.

### Immunofluorescence and Microscopy

Indirect immunofluorescence was performed after fixation of the cells with 3.7% formalin in PBS for 10 min, permeabilization with 0.1% Triton X-100 in PBS for 5 min, and saturation with 2% BSA in PBS, followed by incubation with primary antibodies diluted with 2% BSA in PBS for 45 min. After three washes in PBS, primary antibodies were revealed with Alexa Fluor® 488- or 546-conjugated goat anti-mouse or anti-rabbit antibodies (Invitrogen). Where indicated, TRITC- (Sigma) or Alexa® Fluor 350- (Molecular probes)-conjugated phalloidin was added to reveal F-actin. DNA was stained using bisbenzimide Hoechst dye (Sigma). Cells were mounted in Mowiol® 40–88 (Sigma) and observed under an Axioplan2/LSM META confocal microscope (Zeiss) using Zeiss 40× IR ACHROPLAN 0.8 W or Zeiss 63× C-Apochromat 1.2 W Korr U-V-I objectives. To ensure that only one fluorochrome was detected at any one time, each channel was imaged sequentially using the multitrack recording module before merging. For fluorescence intensity profiles, images were processed with ImageJ using the “plot profile” function, which expresses pixel intensity as height in two-dimensional plots.

**Figure 4:**
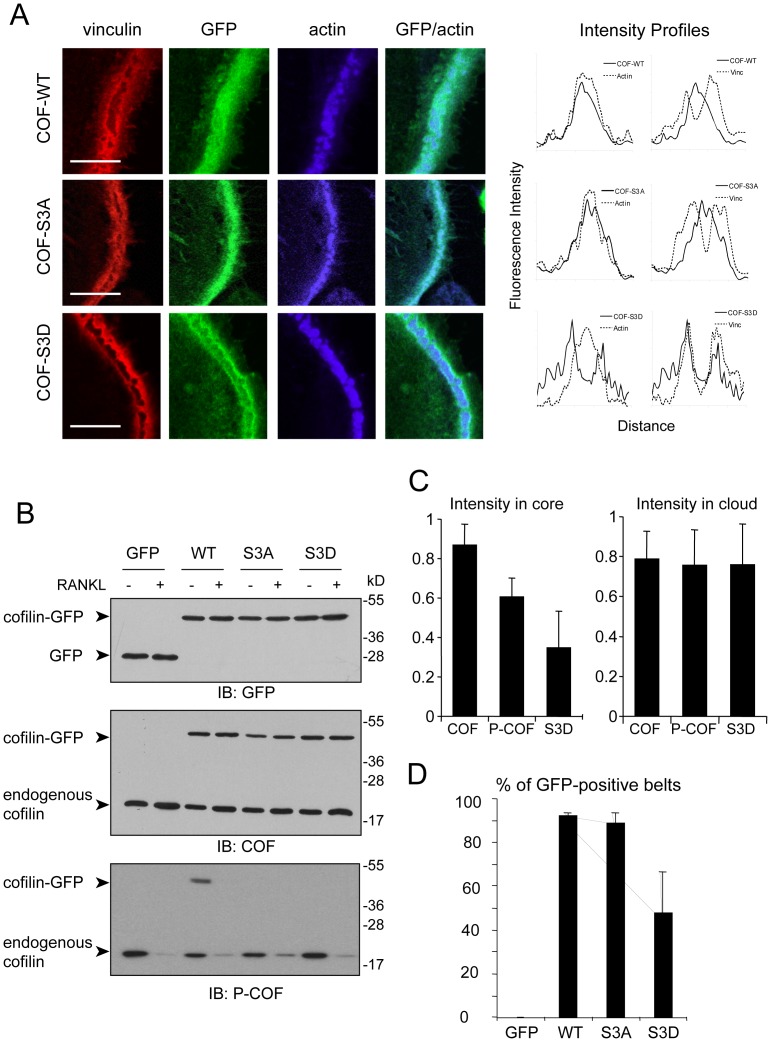
The phosphomimetic mutant COF-S3D is excluded from the core. BMMs were infected with viral supernatants encoding GFP-tagged Cofilin-WT, -S3A and -S3D, then differentiated into OCs. (A) OCs were fixed and stained for vinculin (red) and actin (blue). Panels on the right are merged images of GFP and actin signals. Scale bars: 5 µm. Right panels: Fluorescence intensity profiles of Cofilin-WT, -S3A or -S3D, together with actin or vinculin, measured across podosome belts. (B) Protein extracts were prepared from infected BMMs stimulated (+) or not (−) by RANKL for 5 days, and equal amounts of proteins were immunoblotted for GFP, cofilin (COF) and phospho-cofilin (P-COF). (C) Relative fluorescence intensity of endogenous cofilin (COF), phosphorylated cofilin (P-COF) and GFP-fused cofilin-S3D (S3D) in the podosome belt core (maximum peak of actin fluorescence intensity) and cloud (maximum peak of vinculin fluorescence intensity), setting to 1 the fluorescence intensity maxima of COF, p-COF and Cofilin-S3D in each profile measured across the podosome belt. Graphs show average and SD of 4 (COF and S3D) or 3 (P-COF) profiles. (D) Infected OCs were observed under an inverted microscope and the percentage of podosome belts displaying a GFP signal was determined to analyze whether or not the constructs were addressed to the belt. Results represent the mean ± S.D. of 2 independent experiments.

### Real-time PCR Analyses

Real time PCR analyses were performed as described earlier [23]. Briefly, RNA extractions were carried out using TRIzol reagent (sigma) according to the manufacturer’s instructions. RNA was quantified by spectrophotometry and cDNA synthesized from 1 µg of total RNA with oligo(dT)20 by using the Superscript II First-Strand Synthesis System for RT-PCR (Invitrogen). Real-time PCR was then performed using 1 µl of cDNA per 25 µl total volume. Primers used to amplify GAPDH and Src have been described previously [22]. SSH1 was amplified using Quantitect® primers from Qiagen. Real-time PCR measures to quantify cDNAs were carried out in triplicate and the 95% confidence limits of the ratios to GAPDH were determined by Student *t-*test.

**Figure 5:**
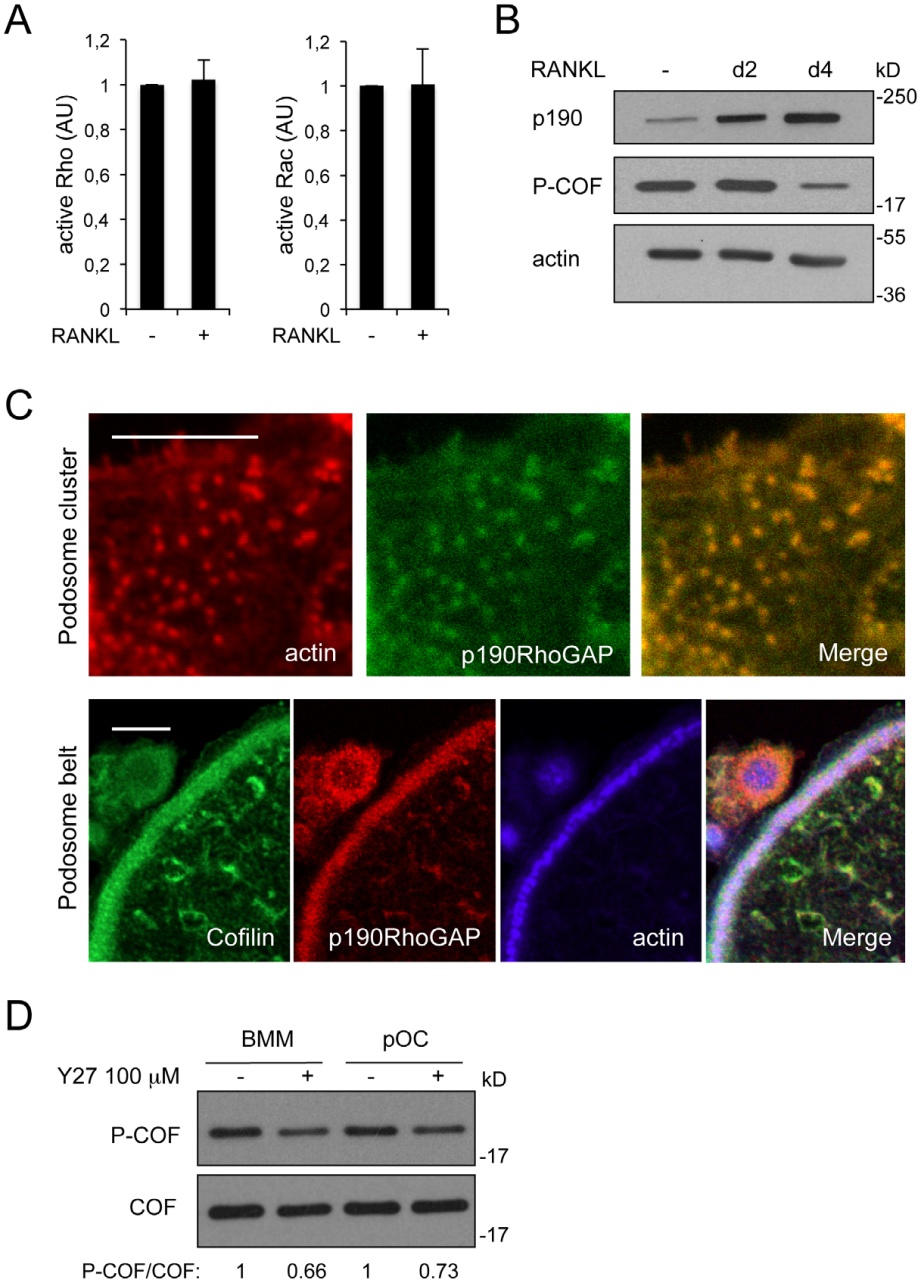
p190RhoGAP is induced by RANKL and targeted to podosomes. (A) RhoA (left panel) and Rac (right panel) activities were measured by G-LISA. BMMs were cultured in the absence (−) or in the presence (+) of RANKL for 3 days. Cell lysates were subjected to G-LISA assays according to manufacturer’s instructions. Shown is a representative of three independent experiments. (B) BMMs were cultured for 4 days in the absence (−) or in the presence of RANKL. Protein extracts were prepared at day 0, 2 and 4 of RANKL addition, and immunoblotted for p190RhoGAP, P-COF and actin as a loading control. (C) OCs differentiated onto coverslips were stained for actin (red) and p190RhoGAP (green) (upper panels) or for cofilin (green), p190RhoGAP (red), and actin (blue) (lower panels). Panels on the right are merged images. Scale bars: 5 µm. (D) BMMs and pre-osteoclasts (pOC) were treated (+) or not (−) for 1 h with 100 µM Y27. Protein extracts were prepared and immunoblotted for phospho-cofilin (P-COF). Total cofilin was revealed after stripping of the phospho-cofilin antibody to serve as loading control. The P-COF/COF ratio was determined by densitometry using the ImageJ software, and the results are shown at the bottom.

### Active RhoA and Rac Quantification

Active RhoA and Rac levels were measured by G-LISA kits according to manufacturer’s instructions (Cytoskeleton). Briefly, 1 mg of total proteins from lysates of BMMs maintained in the absence of RANKL or OCs cultivated with RANKL for 3 days were used to pull down GTP-bound Rho or Rac.

**Figure 6:**
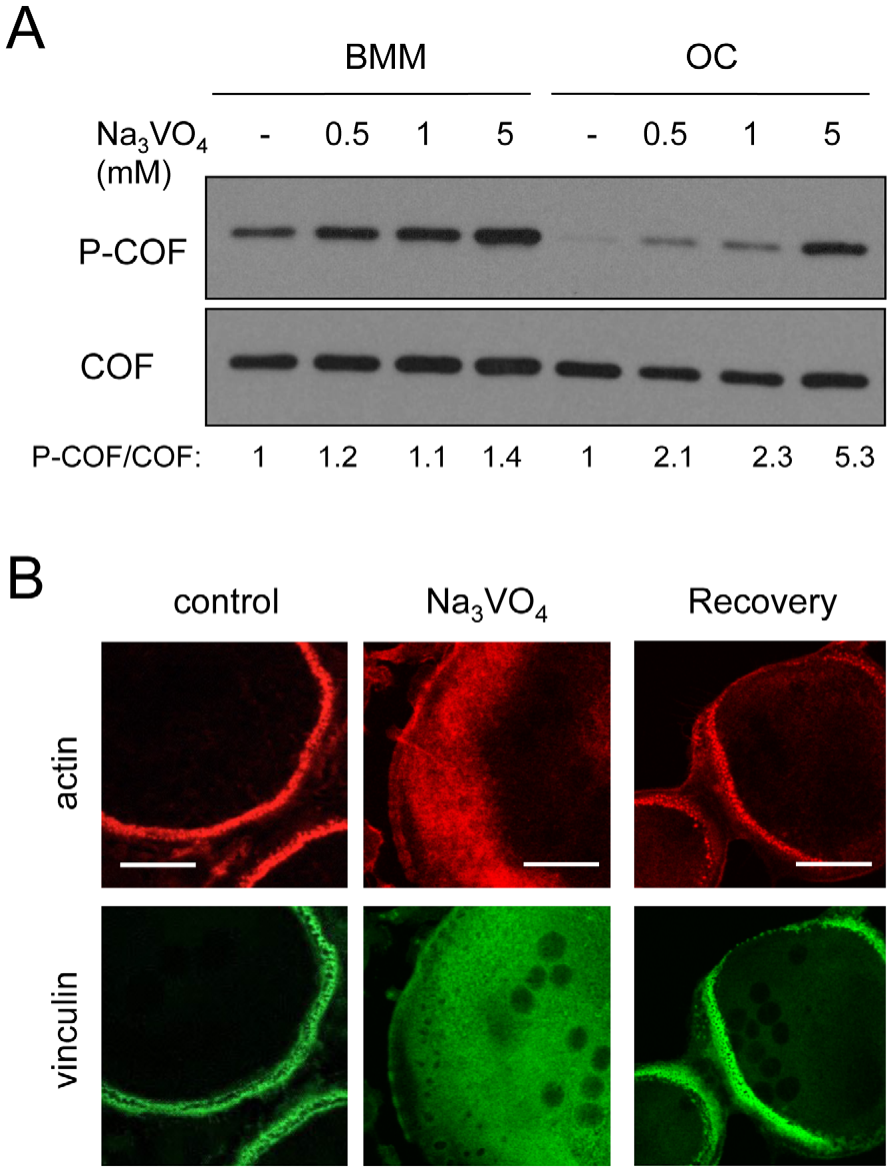
Phosphatases inhibition leads to the concomitant inactivation of cofilin and disassembly of podosomes. (A) BMMs cultured in the absence of RANKL (BMM), or differentiated in the presence of RANKL (OC) for 3 days were incubated for 30 minutes with various amounts of Na_3_VO_4_ (mM). Protein extracts were prepared and equal amounts of proteins were immunoblotted for phospho-cofilin (P-COF). Total cofilin was revealed after stripping of the phospho-cofilin antibody to serve as loading control. A densitometric analysis was performed using ImageJ, and the results of the P-COF/COF ratio is shown at the bottom. (B) OCs were treated by 1 mM Na_3_VO_4_ for 30 minutes then fixed. Alternatively, Na_3_VO_4_ was washed-out and the cells were allowed to recover (Recovery) for 5 h at 37°C. Control and treated OCs were stained for actin (red) and vinculin (green) to check for the presence of podosomes. Note the disassembly of the podosome belt under Na_3_VO_4_ treatment, and its reassembly following the recovery procedure. Scale bars: 10 µm.

### Western Blots

Whole cell extracts were prepared in Laemmli sample buffer, resolved on SDS-PAGE and electrotransferred on nitrocellulose membranes. Immunoblotting was performed according to manufacturer’s instructions and signals were visualized by the ECL Western Lightning Plus detection system (Perkin Elmer) with horseradish peroxidase-conjugated secondary antibodies (GE Healthcare). For densitometry, the amount of cofilin phosphorylation was quantified using ImageJ and was normalized against the total cofilin levels.

**Figure 7:**
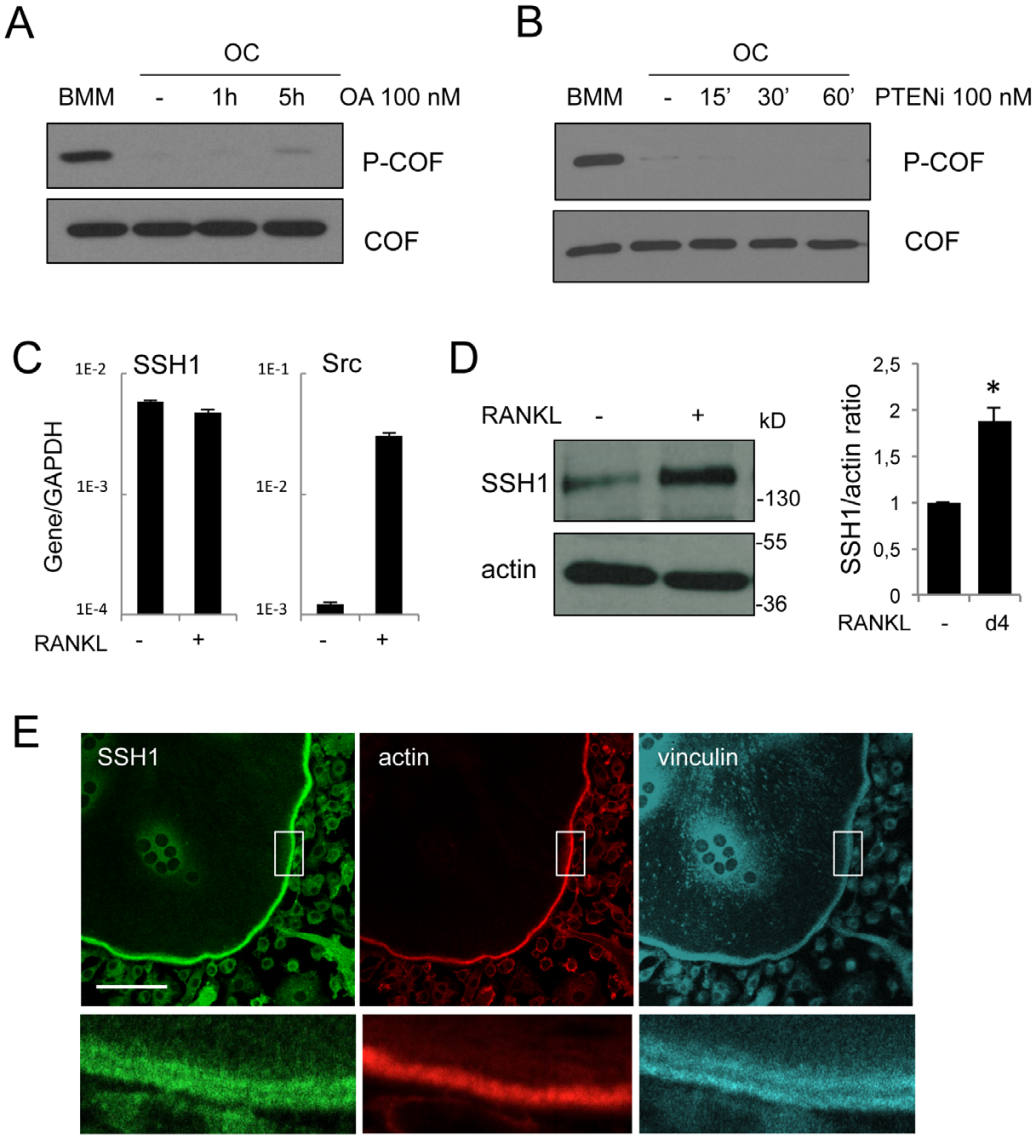
RANKL induces SSH1 protein expression and accumulation in podosome belts. OCs on day 4 of differentiation were treated or not (−) by 100 nM of okadaic acid for 1 or 5 h (A) or 100 nM of PTEN inhibitor (PTENi) for 15, 30 or 60 minutes (B). Protein extracts were prepared and equal amounts of proteins were immunoblotted for phospho-cofilin (P-COF). Total cofilin was revealed after stripping of the phospho-cofilin antibody to serve as loading control. BMM extracts were loaded to show the initial level of phospho-cofilin. (C−D) BMMs were cultured for 4 days in the absence (−) or in the presence (+) of RANKL. Total RNAs were prepared and mRNA levels of SSH1 and Src, relative to GAPDH, were determined by real-time PCR. Results are representative of four independent experiments (C). Protein extracts were resolved by SDS-PAGE and immunoblotted for SSH1 and actin as a loading control. Right panel: densitometric analysis of the ratio of SSH1 to actin levels in unstimulated BMMs (−), versus OCs placed in RANKL for 4 days (d4) (*, *p*<0,001, *t*-test, *n* = 4). Data represent the mean ± S.D. (D). (E) OCs differentiated onto coverslips were stained for SSH1 (green), actin (red), and vinculin (blue). Images at the bottom are magnifications of the boxed regions in the top images. Scale bar: 20 µm.

### Retroviral Infection of BMMs

Cofilin(WT), Cofilin(S3A) and Cofilin(S3D) were obtained by PCR using the pcDNA3/Flag-CofilinS3A vector [24] as a template. PCR-mediated mutagenesis was used to mutate alanine residue 3 (S3A) into a serine residue (to generate the wild-type form of cofilin) or a glutamic residue (to generate the S3D mutant). Amplified fragments were subcloned in pEGFP-N3 (BD Biosciences) between the EcoRI/BamHI sites. EcoRI/NotI fragments were then digested and inserted into the pMXS-puro vector between the EcoRI/NotI sites. For retrovirus production, pMXS vectors containing GFP alone, GFP-Cofilin(WT), GFP-Cofilin(S3A) or GFP-Cofilin(S3D) were transfected into Plat-E cells using Jet-PEI (Ozyme). BMMs were cultured with viral supernatants for 24 h and then selected for expression of the construct with 3 µg/ml puromycine for 3 days. Puromycin-resistant BMMs were used for osteoclast differentiation.

## Results

### Cofilin Activation during Osteoclastogenesis Correlates with the Maturation of Podosome Arrays into Belts

The formation of podosomes and their subsequent organization into clusters, rings and belts is a dynamic process accompanying OC cell differentiation, and supported by strong actin reorganization [7]. Cofilin is an actin severing protein that has been involved in the regulation of several actin-based structures, among which lamellipodia and invadopodia [12]. Its activity is tightly controlled in part by blocking cofilin’s ability to bind actin via serine 3 phosphorylation [11]. Therefore, we thought to determine whether cofilin is activated during osteoclastic differentiation.

Primary OCs were generated by stimulating mouse bone marrow-derived macrophages (BMMs) with M-CSF and RANKL, and the organization of the actin network was recorded in parallel with cofilin activation. Whereas BMMs display small focal complexes (data not shown), the addition of RANKL induces the formation of podosomes, typically at day 2 of differentiation (Figure 1A). From that point, the percentage of cells displaying podosomes increases with time, reaching 40 to 60% of the cells by day 3 (Figure 1A). In parallel, a process of maturation allows podosomes to evolve from clusters of podosomes dispersed throughout the cell, to podosome rings that ultimately form podosome belts at the cell periphery [7]. At day 2, more than 60% of OCs displayed podosome clusters (Figure 1B), and at this time, the level of phosphorylated, inactive cofilin remained unchanged compared to control cells (Figure 1C). Podosome belts accumulated at day 3 (Figure 1B), and this maturation of podosome arrays into belts was associated with a strong reduction in the phospho-cofilin level which dropped to 30% (Figure 1C), thus revealing an activation of cofilin at this stage of osteoclastogenesis. This result demonstrates that cofilin is activated during the process of osteoclast differentiation, and suggests that cofilin activation is linked to podosome maturation into belts rather than to podosome formation.

Formation of the podosome belt is under the control of microtubules, since their disruption causes the collapse of the belt [25], [26]. We transiently depolymerize microtubules in mature OCs and assessed the level of phospho-cofilin in these conditions. As expected, treatment of OCs by nocodazole led to the concomitant disassembly of microtubules and podosome belts (Figure 2A). Under these conditions, we observed a significant increase in the level of phospho-cofilin (Figure 2B). The nocodazole was then washed out and the OCs further incubated for 20 h in fresh medium. The phospho-cofilin level dropped dramatically (Figure 2B), while podosome belts reassembled together with microtubules (Figure 2A). This experiment confirmed the correlation between cofilin activation and the formation of podosome belts during OC cell differentiation.

### Cofilin is Activated in Podosome Belt Cores

The podosome architecture is characterized by an actin-rich core, extending perpendicularly to the substrate, surrounded by a cloud in which several adhesion and signaling proteins accumulate, among which vinculin [27].

To get some insight into the spatial activation of cofilin, we performed indirect immunofluorescence and confocal microscopy using a cofilin antibody that recognizes both the phosphorylated and the non-phosphorylated form, and a phospho-specific antibody directed against Ser-3. On glass, mature OCs are characterized by the presence of a podosome belt at the cell periphery. Confocal analysis of cofilin subcellular distribution revealed that cofilin was enriched in the podosome belt, where it co-localized with both actin and vinculin (Figure 3A and 3D), demonstrating the presence of cofilin in both the actin core and the cloud. By contrast, we found that cofilin was poorly localized to individual podosomes, either in clusters (Figure 3B) or rings (not shown), supporting our hypothesis that cofilin is not involved in podosome formation. The phospho-cofilin signal was also focused in podosome belts (Figure 3C), but displayed a very specific spatial distribution compared to total cofilin. The phospho-cofilin signal was mostly excluded from the core and concentrated in the cloud as demonstrated by the co-localization with vinculin but not actin (Figure 3C). Fluorescence intensity profiles of the phospho- versus total cofilin signals confirmed these observations (Figure 3D). These data establish cofilin as a new component of mature OC adhesion structures, and demonstrate that in podosome belts, cofilin activity is restricted to within the actin core.

To further demonstrate that the phosphorylation status of cofilin impinges on its localization, we infected BMMs with GFP-tagged mutants of cofilin, namely wild-type cofilin (COF-WT), the phospho-mimetic mutant S3D (COF-S3D), or the phospho-deficient mutant S3A (COF-S3A). Similar to the endogenous cofilin, COF-WT and COF-S3A were targeted to podosome belts, where they accumulated in the core (Figure 4A, upper and middle panels). However, by contrast with the endogenous cofilin, COF-WT and COF-S3A were poorly found in the cloud (Figure 4A, intensity profiles, right panels). This probably reflects the constitutive activation of the S3A mutant and the complete activation of COF-WT under RANKL treatment, whereas in all our experiments only a pool of the endogenous cofilin was dephosphorylated hence activated (see Figures 1C and 2B). This hypothesis was confirmed by Western-blot: under RANKL treatment, the COF-WT construct was never detected with the phospho-specific cofilin antibody, whereas endogenous cofilin was (Figure 4B, P-COF immunoblot). By contrast, the mutant COF-S3D accumulated outside the podosome core, in the vinculin-rich cloud (Figure 4A, lower panels and intensity profiles). We further calculated the relative fluorescence levels of total and phosphorylated endogenous cofilin and of GFP-fused cofilin-S3D at the podosome belt core (defined as the peak of actin signal) and the podosome belt cloud (defined as the peak of vinculin signal). As compared to total cofilin, we found that the levels of P-COF and cofilin-S3D were reduced in the core region whereas they were similar in the cloud region of the podosome belt (Figure 4C). This confirms that the inactive form of cofilin tends to be excluded from the core region of the podosome belt.

In addition, we found that the COF-S3D mutant was not targeted to podosome belt as efficiently as COF-WT or COF-S3A (Figure 4D), thus suggesting that the dynamics of cofilin phosphorylation/dephosphorylation is important for its localization. All constructs were expressed at the same level and led to the expression of GFP-tagged cofilin at a level similar to the endogenous cofilin (Figure 4B, GFP and cofilin immunoblots). As expected, only the COF-WT construct was phosphorylated, and this phosphorylation disappeared in response to RANKL (Figure 4B, P-COF immunoblot). The endogenous cofilin was activated in response to RANKL in all conditions (Figure 4B, P-COF immunoblot), showing that in our cellular system the COF-S3D mutant was not dominant-negative over the endogenous cofilin. Consistently, OCs expressing the various forms of GFP-cofilin did not display any obvious phenotype, compared to GFP only (data not shown). Altogether, these analyses revealed that cofilin is activated in podosome cores at the time of belt assembly.

### Cofilin Dephosphorylation Results from the Activation of a Cofilin Phosphatase by RANKL

Cofilin activation in invadopodia was recently linked to the local inactivation of Rho family members, achieved by local targeting of Rho GEFs and GAPs [19]. To investigate such a mechanism in OCs, we first analyzed Rho and Rac activities throughout OC differentiation. We found that cofilin is activated by RANKL without changes in global Rho-GTP and Rac-GTP levels (Figure 5A). However, we found that the expression of p190RhoGAP is strongly induced by RANKL (Figure 5B). Furthermore, p190RhoGAP is present in individual podosomes (Figure 5C, upper panels), and concentrated in podosome belts where it colocalized with both actin and cofilin (Figure 5C, lower panels). Taken together, these results suggest that despite a stable global activity of Rho/Rac throughout the process of OC differentiation, a local decrease in Rho activity might be achieved by the local accumulation of p190RhoGAP in podosome cores. In invadopodia, the local accumulation of p190RhoGAP leads to an activation of cofilin by modulating the RhoC/ROCK/LIMK pathway [19]. In BMMs and OC precursors (i.e. BMMs stimulated by RANKL for two days but still mononucleated), high doses of the ROCK inhibitors Y27 (100 µM) or H-1152 (10 µM) are required to achieve a mere 25% reduction in phospho-cofilin levels (Figure 5D and data not shown). This result shows that the phosphorylation of cofilin in OC precursors is unlikely controlled by a ROCK-dependent pathway. Therefore, modulation of this pathway by p190RhoGAP can not be the mechanism for cofilin activation upon RANKL treatment.

Cofilin activity is held in balance by the opposing action of kinases that inactivate it and phosphatases that activate it. Given the strong dephosphorylation of cofilin observed by Western-blot (see Figure 1C), we investigated whether a phosphatase could play a role in the activation of cofilin in OC. To this end, BMMs and OCs were treated by Na_3_VO_4_, a broad phosphatase inhibitor. Whereas cofilin phosphorylation was almost insensitive to Na_3_VO_4_ in BMMs maintained in the absence of RANKL, the phospho-cofilin level increased up to 5-fold in OCs (Figure 6A, densitometric analysis). In addition, this increase in cofilin phosphorylation was associated with a disassembly of podosomes upon Na_3_VO_4_ treatment (Figure 6B). This disassembly of podosomes did not result from a toxic effect of Na_3_VO_4_ since OCs were able to reassemble podosome belts when the drug was washed-out (Figure 6B, right panel). Although the use of Na_3_VO_4_ alone is not sufficient to demonstrate that cofilin activation is directly associated with a phosphatase, it implies a RANKL-induced phosphatase activity in the pathway leading to cofilin activation.

### The Phosphatase SSH1 is a Podosome Belt Component Induced by RANKL

Among the phosphatases that have been involved in the regulation of cofilin [16], members of the Slingshot family were described as insensitive to most phosphatase inhibitors other than vanadate [14], [28]. In addition, cofilin dephosphorylation in OCs was not inhibited by the PP1/PP2A inhibitor okadaic acid (Figure 7A), nor by inhibition of PTEN (Figure 7B), two phosphatases that have been involved in cofilin activation in other cell systems [29], [17]. This prompted us to test the involvement of SSH members in cofilin activation in OC. We found that RANKL does not regulate expression of the SSH1 mRNA, by contrast with Src which expression is strongly induced (Figure 7C). However, SSH1 level of expression is significantly increased by RANKL at the protein level (Figure 7D). An analysis by confocal microscopy revealed the accumulation of SSH1 in the podosome belt (Figure 7E). The staining appeared as two bands flanking the actin signal, a pattern similar to the P-COF signal described in Figure 3C. The accumulation of SSH1 in podosome belts strongly suggests that this phosphatase might contribute to the local activation of cofilin in this region.

The induction of SSH1 expression in response to RANKL, together with the co-localization between SSH1 and its known substrate phospho-cofilin in podosome belts, strongly suggest that SSH1 is responsible for the activation of cofilin during OC differentiation.

## Discussion

The well-characterized functions of cofilin in actin remodeling prompted us to check its involvement in osteoclastic podosome, a dynamic cytoskeletal structure that undergoes strong reorganization associated with high turnover during osteoclast differentiation.

We performed a detailed analysis of the spatial and temporal regulation of cofilin activity. The results presented herein revealed that cofilin is present in podosome belts, the adhesion structure characteristic of mature OCs. In podosome belts, the relative distribution of total cofilin and phospho-cofilin demonstrated that the active form of cofilin is concentrated in the actin-rich core. These results are consistent with biochemical studies showing that only the de-phosphorylated form of cofilin binds to actin [30]. This result is also in agreement with a recent report describing the presence of cofilin in the core of invadopodia, whereas phospho-cofilin is concentrated outside [19]. Our study demonstrates that cofilin is present and activated in mature OC adhesion structures but not in individual podosomes. This suggests a function for cofilin in podosome maturation rather than formation. Consistently, cofilin silencing in invadopodia does not prevent the assembly of invadopodia, but impacts on their stability [18]. The actin-severing and capping protein gelsolin is another actin-cytoskeleton regulating protein that has been localized to podosomes in several cell types. Silencing of gelsolin blocked the formation of podosomes in OCs [31], but not in dendritic cells [32]. It is therefore possible that the processes of podosome assembly and maturation require different actin-severing proteins respectively.

The formation and dynamics of podosomes have been linked to a local decrease in actomyosin contractility, under the control of Rho GTPases [33], [8]. Similarly, a recent study described a decrease in RhoC activity at invadopodia, mediated by a local targeting of Rho GEFs and GAPs [19]. In agreement, we found that p190RhoGAP expression is regulated by RANKL, and that the protein is targeted to podosomes in OCs (Figure 5). This raises the hypothesis that the activity of Rho family members is locally regulated by p190RhoGAP in osteoclastic podosomes, as in A7r5 cell podosomes and invadopodia [33], [19]. In invadopodia, this pathway was further linked to the regulation of cofilin phosphorylation, by a cascade involving p190RhoGAP/RhoC/ROCK and LIMK [19]. In OCs, this pathway is unlikely to control cofilin activation. First, p190RhoGAP expression is induced by RANKL before cofilin activation (Figure 5B). Second, p190RhoGAP is present in individual podosomes, but cofilin is not (Figure 5C). And third, ROCK does not appear as a major kinase for the regulation of cofilin activity in OCs, since cofilin phosphorylation in BMMs is poorly sensitive to ROCK inhibition (Figure 5D). This does not preclude a role for p190RhoGAP in podosome assembly and/or organization in OCs, but makes it unlikely to be a major pathway for the regulation of cofilin activity at podosome belts. Induction of p190RhoGAP might rather be required to regulate Rho family members activity at the onset of podosome assembly, as in vascular smooth muscle cells [34].

To determine the molecular basis for cofilin dephosphorylation induced by RANKL, we investigated another hypothesis: activation of a phosphatase. Among the phosphatases that have been described to target cofilin, we found that the expression of SSH1, the founding member of the Slingshot family [14], is upregulated by RANKL. Furthermore, we ruled out the implication of PP1, PP2A and PTEN by the use of specific inhibitors. Therefore we went on to analyze SSH1 involvement in cofilin activation in OCs. Similar to cofilin, SSH1 was found in mature OCs adhesion structures, i.e. podosome belts (Figure 7E). Since SSH activity is increased by high cellular F-actin levels [14], [35], the presence of SSH1 in the podosome belt might reflect its activation by F-actin binding. The proximity between SSH1 and its substrate, phospho-cofilin, would then result in cofilin activation specifically in this subcellular compartment. In other hematopoietic cells, the regulation of cofilin activity has been involved in neutrophil polarization and chemotaxis under the control of SSH2 [36], whereas cofilin activation mediated by PTEN regulated macrophage phagocytosis of fungi [17]. However, the role of PTEN in cofilin activation is somehow controversial since PTEN had been previously reported to inhibit cofilin activity by decreasing the effects of SSH1 [37]. In agreement, a recent study showed that the loss of PTEN activates cofilin [38]. Cell-type specific signaling pathways may account for these differences. A study has reported the regulation of SSH1 translation via elongation of its poly(A) tail, in mammary cells overexpressing Aurora-A [39]. Similarly, we showed that RANKL increases SSH1 expression by a post-transcriptional mechanism (Figures 7C and D). Further analyses are required to unravel the mechanism of RANKL-induced expression of SSH1 in OCs.

Podosomes are dynamic and short-lived structures with an average life span of 1–5 min [4], [40], [41]. Interestingly, as podosomes become associated in belts, their life span is reduced and they become further destabilized [42], [43]. This increased turnover of podosomes in belts involves the signaling activities of Src, dynamin, Pyk2 and cyt-PTPe [40], [43]–[45], but the underlying effectors are still unknown. Our results demonstrate the presence of the active form of cofilin in the core of podosomes, only when assembled in belts. This raises the hypothesis that the severing activity of cofilin is the effector mechanism by which the life span of podosomal cores is shortened in belts. Based on the model described by Oser and Condeelis [12], the first step in cofilin activation at podosome belts would be the dephosphorylation by SSH. At this step, cofilin remains inactive in the F-actin compartment by binding to cortactin. The phosphorylation of cortactin, most probably by Src, would then release an active cofilin to generate free barbed ends for actin remodeling within podosome cores. By activating Src, Cyt-PTPe could participate in this pathway [43]. In addition, cortactin tyrosine phosphorylation activates dynamin-II’s GTPase activity, which remodels actin filaments making them more accessible to cofilin [46].

Together with other studies [18], our work excludes cofilin as a regulator of podosome assembly, and rather involves its severing activity in osteoclastic podosome organization. Given the well-established links between the organization of podosomes in OCs and the ability of these cells to degrade bone, cofilin function in OCs deserves further studies as it may constitute a target to control OC activity.
